# Association between the Number of Consecutively Scheduled Telehealth Visits and Video Usage

**DOI:** 10.1089/tmr.2024.0014

**Published:** 2024-08-02

**Authors:** Kenan Katranji, Shruti Bakare, Sarah Rose Cass, Helena Mirzoyan, Hannah B. Jackson, Christine Zhang, Kevin Chen

**Affiliations:** ^1^ Office of Ambulatory Care and Population Health, New York City Health + Hospitals, New York, New York, USA.; ^2^ Division of General Internal Medicine and Clinical Innovation, New York University Grossman School of Medicine, New York, New York, USA.

**Keywords:** telehealth, primary care, schedules, administration

## Abstract

**Background:**

Schedule design may contribute to successful completion of synchronous telehealth visits by video (versus audio-only). Clustering telehealth visits on schedules may minimize workflow inefficiencies.

**Methods:**

We analyzed data from 21 primary care sites in an urban public health care system from March 1 to September 30, 2022. We used linear regression to test for associations between the number of consecutive telehealth visits scheduled per clinicians’ half-day sessions (1 to 9+) and the proportion of telehealth visits scheduled and, separately, completed as video (versus audio-only).

**Results:**

For each additional consecutive telehealth visit scheduled, there was a 6.85% [95% confidence interval 4.80 − 8.90%] increase in the absolute percentage of visits scheduled as video visits. For each additional consecutive telehealth visit scheduled, there was a 2.88% [0.59 − 5.18%] increase in the absolute percentage of visits completed as video visits.

**Conclusions:**

Clustered telehealth visits are positively associated with scheduling and completion of telehealth visits by video.

## Introduction

For many safety net settings, provision of synchronous telehealth visits by both audio-only and video has been important for promoting equitable access to telehealth, particularly during the 2019 coronavirus pandemic. However, consistent use of video visits has been important for health systems looking to leverage the convenience of telehealth, while benefiting from enhanced clinical capabilities (e.g., limited physical or environmental examination), patient experience, and reimbursement provided by video versus audio-only visits.^1–3^ Although elements such as patients’ access to appropriate technology and digital literacy are also critical for enabling successful video visit participation, provider and facility influences may be more associated with video visit completion than patient-level characteristics.^4,5^ This suggests that factors such as provider workflows may contribute to whether a scheduled telehealth visit is completed as video or audio-only. If a clinician’s schedule has alternating in-person and telehealth visits, there may be workflow inefficiencies in switching between modalities or differences in prioritization when some patients are physically present and others are not.^6^

We hypothesized that consecutive telehealth visits could reduce some of these frictional elements. We investigated associations between the number of consecutive scheduled telehealth visits and the rate of successful completion of these visits by video in safety net primary care clinics, offering both modes (video and audio-only) of telehealth.

## Methods

### Setting and data source

We analyzed electronic health record (EHR) scheduling data across 21 adult primary care sites in a large, urban public health care system in the northeastern United States from March 1, 2022, to September 30, 2022. De-identified data were collected on May 25, 2023. During this period, visits could be scheduled as in-person, audio-only, or video visits based on patients’ and clinicians’ preferences and determination of appropriateness. Patients could schedule appointments at the clinic, by calling a centralized call center, or through online self-scheduling using a patient portal (<5% of visits of any type were scheduled online). All clinics utilized a shared EHR with a common embedded video visit platform. Patients with scheduled video visits are instructed to access the video visit using their patient portal account 15 minutes before the scheduled time. If a patient is not present in the video instance at the time of visit, the recommended operating procedure is for staff to reach out to the patient by text message, email, and/or phone call to facilitate a video connection. If there is no response to the video invitation or the patient declines verbally, then staff may opt for conducting the visit by audio-only. For this analysis, we included only visits scheduled to occur by audio-only or video.

Visits were classified as having been scheduled and, separately, completed by audio-only or video. The mode for which a visit was scheduled was determined using the EHR’s appointment-type label selected at the point of scheduling. The mode by which the visit was ultimately completed was identified using billing (current procedural terminology) codes. Visits with codes 99441–99443 were classified as having been completed by audio-only. Visits with any other code plus a telehealth modifier (95, GT) were classified as video. Visits could be scheduled as one type of visit and completed as another. For example, a visit could be scheduled as a video visit but billed (completed) as having been completed by audio-only; the reverse is also possible.

### Primary outcomes

The primary outcomes were as follows: (1) the percentage of telehealth visits scheduled as video (versus audio-only) and (2) the percentage of scheduled telehealth visits completed as video (versus audio-only) visits. We use these outcomes as proxies for how well provider workflow enables clinicians to conduct video visits.

### Primary exposure

The primary exposure was the number of consecutive telehealth visits scheduled on clinicians’ schedules. A typical primary care half-day clinical session in the system has 8–10 appointment slots. We created strata based on the number of consecutive telehealth visits scheduled (1 to 9+). Generally, due to the way in which scheduling and billing are conducted, no billable telehealth visit occurs outside of sessions when clinicians are assigned to be clinically active.

### Statistical analysis

We examined percentages of visits scheduled and completed as video visits in each stratum of consecutive telehealth visits. We used two separate unadjusted ordinary least squares linear regression models to quantify the association between the number of consecutive telehealth visits (independent variable) and each primary outcome (dependent variable).

We used Stata SE, version 15 (StataCorp), for all analyses. The *regress* command was used for linear regression.

This study was exempted from full review by the Biomedical Research Alliance of New York with a waiver of informed consent to access anonymized medical record data.

## Results

We analyzed 73,022 scheduled telehealth (audio-only and video) visits by 569 clinicians. The distribution of consecutive telehealth visits is described in Table 1.

**Table 1.** Frequency of Consecutive Telehealth Visits and Percentage Scheduled and Completed as Video Visits

Number of consecutive telehealth visits	Number of clinicians with clusters	Number of scheduled telehealth (audio-only and video) visits within clusters	% Consecutive telehealth visits scheduled as video visits	% Consecutive telehealth visits completed as video visits
1	174	5,597	27%	10%
2	98	7,027	35%	16%
3	60	5,370	58%	21%
4	46	5,574	58%	16%
5	38	7,880	54%	14%
6	27	4,352	60%	12%
7	18	5,002	69%	28%
8	43	9,806	80%	25%
9+	65	22,414	90%	44%

**FIG. 1.**
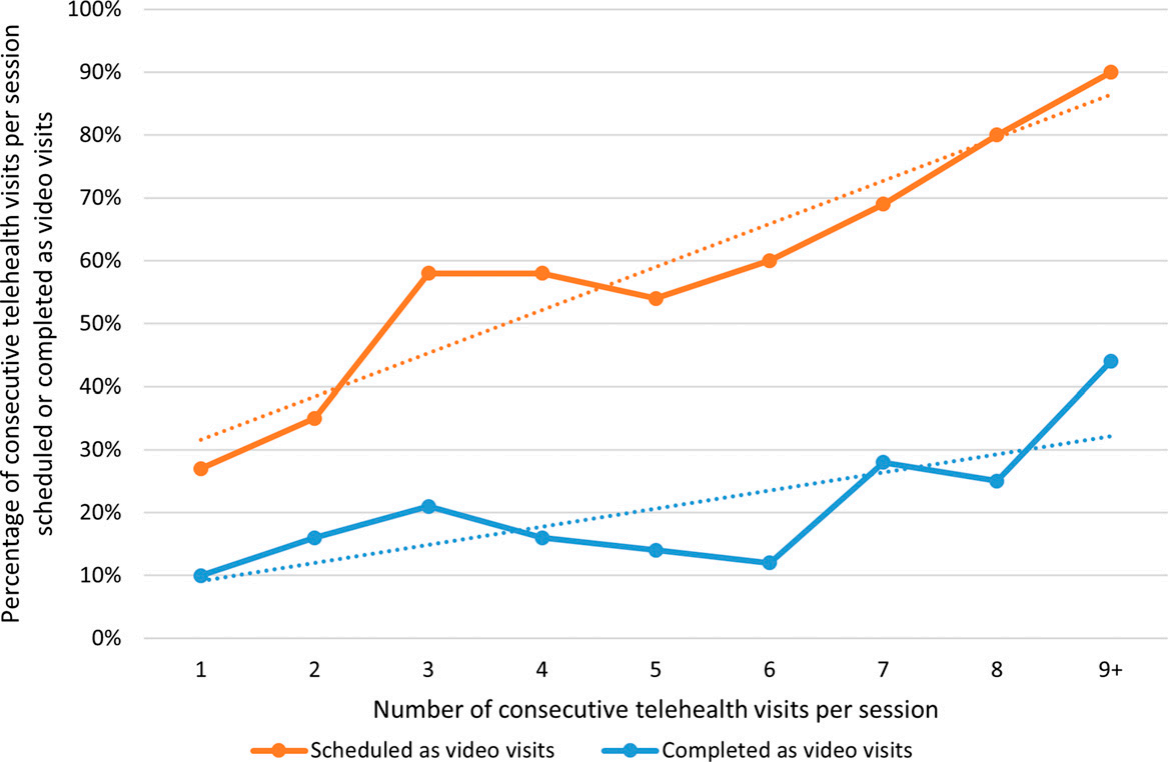
Percentage of telehealth visits scheduled and completed as video visits by number of consecutive telehealth visits.

Telehealth visits scheduled consecutively were more likely to be scheduled and, separately, completed as video (as opposed to audio-only) visits (Fig. 1). For each additional consecutive telehealth visit scheduled, there was a 6.85% (95% confidence interval 4.80 − 8.90%) (*p* < 0.01) increase in the absolute percentage of visits scheduled as video visits. For each additional consecutive telehealth visit scheduled, there was a 2.88% (0.59 − 5.18%) (*p* = 0.02) increase in the absolute percentage of visits completed as video visits.

## Discussion

The number of consecutive telehealth visits scheduled was positively associated with the percentage of telehealth visits scheduled and completed as video (versus audio-only) visits. Although causality cannot be determined from this observational study, this pattern is consistent with our hypothesis that consecutively scheduled telehealth visits may reduce frictional aspects of toggling between in-person and telehealth visits. These aspects may include the following: minimizing clinical workflow interruptions, limiting decision fatigue (and reducing likelihood of relying on “easier” choices) from needing to prioritize in-person and virtual patients, and reducing reluctance to attempt connecting with patients by video (or troubleshooting technology) with fewer competing demands.^7,8^

Our linear model estimates that with 9+ consecutive telehealth visits in a session, the scheduling rate for video versus audio-only visits would be more than double (86% vs. 32%) and the completion rate by video would be over triple that (32% vs. 9%) of single telehealth visits. For systems offering both audio-only and video visits, the percentage of video visits may not be optimally 100%, as audio-only visits may play an important role in equitable access for telehealth services, particularly in safety net settings.^4,5^ Nonetheless, the gap between the scheduled and completed video visits at every level of consecutiveness suggests further work to be done in supporting patients and clinicians in identifying the most appropriate visit modality and completing visits using the scheduled modality. While the absolute percentage difference in video completion at the highest level of consecutive visits may not be very impressive, each visit completed by video instead of audio-only could represent a financially important difference in per-visit reimbursement based on historical differences in video versus audio-only payments across all payors. At the time of writing, payment parity for telehealth enacted during the 2019 coronavirus pandemic was largely suspended for nonbehavioral health services at the end of the public health emergency. Continued financial support for audio-only telehealth services is uncertain. Ongoing payment parity for audio-only, video, and in-person visits may obviate reimbursement-related pressures for prioritizing video over audio-only completion. Of course, comparative clinical safety and efficacy of all three modes should be studied as well to inform this. Pending policy changes, health systems looking to encourage successful completion of telehealth visits by video may consider scheduling telehealth visits in consecutive blocks as one part of a multi-pronged approach.

### Limitations

We did not control for patient, clinician, or other factors that may affect propensity to schedule or complete telehealth visits by video over audio-only or to have consecutive telehealth visits. For example, clinicians who have entire half-day sessions of telehealth may have differing circumstances and comfort with telehealth than clinicians who have hybrid sessions with interspersed telehealth visits. Findings may mainly be relevant for health systems that offer both audio-only and video visits.

Nonetheless, our findings provide data in support of utilizing consecutively scheduled telehealth visits as a mechanism for improving video visit scheduling and completion.
